# Injuries and complaints in the Brazilian national volleyball male team: a case study

**DOI:** 10.1186/s13102-023-00687-3

**Published:** 2023-07-04

**Authors:** Alessandro Haupenthal, Thainá Bufon, Matheus Cardoso dos Santos, Luiza Marx Matte, Elisa Dell’Antonio, Felipe Malzac Franco, Ney Coutinho Pecegueiro do Amaral, Lucas dos Santos Costa, Guilherme S. Nunes

**Affiliations:** 1 Department of Health Sciences, Federal University of Santa Catarina, Araranguá, SC Brazil; 2 Clínica Personal Fisio, Florianópolis, SC Brazil; 3 Healthcare Department, Brazilian Volleyball Confederation, Rio de Janeiro, RJ Brazil; 4 Department of Physiotherapy and Rehabilitation, Federal University of Santa Maria, Av. Roraima, 1000, Santa Maria, RS, CEP 97105-900 Brazil; 5 College of Health and Sport Science, Santa Catarina State University, Florianópolis, SC Brazil

**Keywords:** Incidence, Prevalence, Sport, Physiotherapy, Prevention

## Abstract

**Background:**

The literature reports a vast amount of epidemiological information on injuries in volleyball athletes. However, little is known about the incidence of injuries in elite athletes of international level participating in major competitions, such as world championships and Olympic games. The objective of the study was to analyse the incidence of injuries in elite professional volleyball athletes, and the prevalence of complaints reported by athletes.

**Methods:**

This is a case study in which data were collected between April 2018 and August 2021. All the athletes called to play for the Brazilian national male volleyball team during the analysis period participated. From the athletes’ medical records, the occurrence of injuries (injurious events that lead to a time off from activities) and complaints (discomforts that did not lead to a time off from activities) were analysed. Frequency data were used to calculate incidence, prevalence and ratios.

**Results:**

From 41 athletes who played for the team during the analysed period, 12 athletes had 28 injuries and 38 athletes reported 402 complaints. For injuries, an incidence of seven injuries/1,000 h of competition and two injuries/1,000 h of training was observed. The average recovery time of the athletes was 10 days. The regions with the highest prevalence of injuries were the knee (111/1,000 athletes) and ankle (69/1,000 athletes). For complaints, 402 complaints required 1,085 treatment sessions, with the regions with the highest prevalence of complaints being the knee (261/1,000 complaints) followed by the shoulders (236/1,000 complaints). Athletes aged above 23 years and those playing as middle blockers and outside hitters presented a higher prevalence of injuries and complaints.

**Conclusions:**

Almost one-third of the athletes had injuries and almost all athletes reported complaints during the study period. Injuries and complaints were more prevalent in the knees. Complaints caused a high demand for the healthcare team. To manage risk of injuries for overload, specific injury prevention strategies are needed and should be included as an essential component of the training plan for elite volleyball players.

## Background

Volleyball is one of the most popular sports in the world, with more than 200 national federations and five continental confederations registered in the International Volleyball Federation (FIVB) [1, 2]. It is considered a safer sport when compared to other team sports, such as football, because of its non-contact characteristic, which decreases the chance of harmful contact between players [3, 4]. However, injuries still occur during volleyball activities [5, 6]. Injuries during volleyball practice are usually related to a high level of physical demand with specific tasks, such as shifting, jumping, and landing, and excessive repetition of movements which may lead to overload [6–8].

In professional volleyball, it is estimated an incidence of 14 injuries per 1,000 h of activity, including training sessions and matches [9]. Previous studies have reported a high incidence of ankle injuries among volleyball athletes [5, 10–12], with ankle sprains accounting for up to half of all acute injuries [6]. Additionally, overuse-related problems affect the knees, back, and shoulders [5]. However, although the literature reports epidemiological information on injuries in volleyball, most studies have presented data from national or amateur-level athletes [3, 4, 6–8, 10–14]. Therefore, the incidence and severity of injuries in elite athletes of international level participating in major competitions, such as world championships and Olympic games, are unclear [5]. Injuries result in short- and long-term social and economic consequences [15], with direct costs related to the treatment and indirect costs related to the time off from sports activities [13].

Complaints of pain are common among elite athlete carriers [16]. Usually, these complaints are not sufficient to prevent the engagement of athletes in training sessions and matches; however, they still require attention from the health professionals. The management of complaints is likely to be as important as that of injuries, considering that complaints potentially indicates risk of injury from overuse [17–19]. Nonetheless, little is known about the complaints of elite volleyball athletes and how complaints may influence health professionals’ activities in these elite teams.

Compared to other volleyball athletes, elite athletes of national teams present specific characteristics, these athletes have greater workloads to achieve excellence and a longer season without proper rest time for recovery. Elite athletes may present injuries and complaints during the international season which were developed during the national season playing for their domestic teams [20]. Knowledge of athletes’ complaints and injuries allows for the development of specific preventive programs, which could be included as an essential component of training plans for volleyball elite athletes [21]. Therefore, in the present study we aimed to analyse (i) the incidence of injuries in the athletes of the Brazilian national male volleyball team, verifying its impact on time off from activities, severity of the injuries, and the most affected anatomical regions; (ii) the prevalence of complaints reported by athletes, verifying its impact on the activity of physiotherapists, the most affected anatomical regions, and the characteristics of these complaints in relation to the affected structures; and (iii) whether the prevalence of injuries and complains differs among athletes of different age groups and positions.

## Methods

### Design

This is a case study which was approved by the Ethics Committee of the Federal University of Santa Maria (CAAE 45324421.2.0000.5346) and was reported based on the STROBE Sports Injury and Illness Surveillance checklist (STROBE-SIIS) [22].

### Participants

We included all athletes called to play for the main team of the Brazilian national volleyball male team between 2018 and 2021, considered able to play volleyball at a competitive level after a health evaluation. Athletes with injuries that occurred prior to the presentation were included if the healthcare team considered that the injury was treatable within a viable space of time.

### Procedures

Injuries and complaints that occurred while the athletes were playing for the National team between April 2018 and August 2021 were considered. The physiotherapy department of the Brazilian male volleyball team recorded the occurrence of injuries and complaints on the healthcare department’s registry platform for all training sessions and matches during the study period.

The data for the present study were extracted from medical records, and the following data were analysed: (i) Number of injuries – an injury was defined as an injury event that caused the athlete to be away from their activities for at least one day [3, 22, 23]; (ii) Diagnostic characteristics of the injuries [22]; (iii) Time away from the activities caused by injuries; (iv) Severity of injuries categorised according to time of absence as light (1–7 days), moderate (8–28 days), and severe (over 29 days) [22]; (v) Number of complaints – a complaint was defined as any discomfort that did not lead the athlete to be absent from activities but required attention from the healthcare team [17, 22] – in cases of complaints in a previously treated anatomical region, it was considered as a new complaint when the need for intervention occurred more than a week apart; (vi) Classification of complaints according to the affected structure in muscle, ligament, joint, tendon or bone [24, 25]; (vii) Number of sessions carried out to treat complaints; (viii) Anatomical regions of injuries and complaints [22, 24, 26]; (ix) Total hours dedicated to training sessions and matches, which were obtained from the technical commission records; and (x) Characteristics of the athletes, including age, weight, height and position.

### Data analysis

Among the analysed period, the year 2020 was disregarded, because there was no activity due to the COVID-19 pandemic. Therefore, the frequency of injuries and complaints for the years 2018, 2019 and 2021 were analysed. The record of workload during training sessions and matches was only available for 2018; this period refers to training and matches for the world championship and Volleyball Nations League.

Data were descriptively analysed using frequencies presented as percentages and absolute values. For the results regarding time away from activities and athletes’ characteristics, the data were presented as measures of central tendency and dispersion. Annual rates of injuries, complaints, and sessions for complaint treatment were obtained using the ratio between absolute values and time in years of follow-up. Furthermore, the annual rate of complaints was standardised by the number of exposed athletes (complaints/athletes/year). Injury incidence for 2018 was calculated by dividing the number of injuries by the total number of hours in the exposed period, presented as the number of injuries per 1,000 h. Thus, the incidence of injury was reported in relation to total time of exposure, including all athletes (regardless the amount of participation in matches) and all activities related to training and matches for the analysed year. The prevalence of injured anatomical regions (95% confidence interval) was calculated by dividing the total number of injuries in the region, by the total number of exposed athletes, presented as the number of injuries per 1,000 athletes. In addition, the prevalence of anatomical regions affected by complaints (95% confidence interval) was calculated by dividing the total number of complaints in the region, by the total number of complaints, presented as the number of complaints in the region per 1,000 complaints. Considering previous reports [4, 5, 27], we also analysed our data by stratifying the cohort in into age groups (athletes aged ≤ 23 years and those above 23 years), and by athletes’ position in order to compare the frequencies of injuries and complaints for each year of analysis. To examine potential differences in frequencies between groups, we used chi-square tests for independence, with a significance level of α = 0.05. When statistical analysis was not possible due to low frequency for some groups, these data were analysed descriptively. All statistical analyses were performed using R software version 4.3.

## Results

Of the 41 athletes who played for the national team in the analysed years, 12 athletes (29%) presented injuries, and 38 athletes (93%) reported complaints that required care from the physiotherapy department (Table 1). The proportion of athletes aged below and above 23 years was 13/16 athletes in 2018 (χ^2^ = 0.31, df = 1, *p* = 0.58), 11/18 athletes in 2019 (χ^2^ = 1.69, df = 1, *p* = 0.19), and 6/20 athletes in 2021 (χ^2^ = 7.54, df = 1, *p* = 0.01) – proportion of athletes between age groups was significantly different in 2021.

**Table 1:** Characteristics of the participants  - data in mean (standard deviation)

		**Age (years)**	**Height** **(m)**	**Weight** **(kg)**	**Athlete Position**
**All years**	**All athletes (*n* = 41)**	26 (5)	1.99 (0.08)	91 (11)	LB (7%), MB (20%), OS (20%), OH (41%), ST (12%)
	**Injured (*n* = 12)**	28 (4)	2.02 (0.08)	98 (12)	LB (0%), MB (33%), OS (17%), OH (33%), ST (17%)
	**Complaints (*n* = 38)**	27 (5)	1.98 (0.09)	92 (11)	LB (7%), MB (17%), OS (20%), OH (37%), ST (12%)
	**No Injury (*n* = 29)**	25 (5)	1.98 (0.08)	89 (10)	LB (11%), MB (14%), OS (21%), OH (46%), ST (7%)
	**No Complain (*n* = 3)**	19, 21, 27	1.94, 2.00, 2.12	79, 81, 104	MB, OH (2)
**2018**	**All athletes (*n* = 29)**	26 (6)	1.99 (0.08)	91 (11)	LB (7%), MB (21%), OS (17%), OH (41%), ST (14%)
	**Injured (*n* = 6)**	28 (4)	2.01 (0.07)	95 (13)	LB (0%), MB (33%), OS (0%), OH (50%), ST (17%)
	**Complaints (*n* = 27)**	26 (6)	1.99 (0.08)	90 (12)	LB (7%), MB (22%), OS (15%), OH (41%), ST (15%)
	**No Injury (*n* = 23)**	25 (6)	1.98 (0.08)	89 (11)	LB (9%), MB (17%), OS (22%), OH (39%), ST (13%)
	**No Complain (*n* = 2)**	23, 25	1.96, 2.02	90, 98	OS, OH
**2019**	**All athletes (*n* = 29)**	25 (4)	1.99 (0.08)	91 (12)	LB (7%), MB (28%), OS (17%), OH (35%), ST (14%)
	**Injured (*n* = 7)**	26 (4)	2.04 (0.10)	102 (11)	LB (0%), MB (57%), OS (14%), OH (14%), ST (14%)
	**Complaints (*n* = 23)**	25 (4)	1.99 (0.08)	91 (12)	LB (9%), MB (22%), OS (17%), OH (39%), ST (13%)
	**No Injury (*n* = 22)**	25 (4)	1.98 (0.07)	88 (10)	LB (9%), MB (18%), OS (18%), OH (41%), ST (14%)
	**No Complain (*n* = 6)**	26 (5)	2.01 (0.08)	93 (13)	LB (0%), MB (50%), OS (17%), OH (17%), ST (17%)
**2021**	**All athletes (*n* = 26)**	27 (5)	1.99 (0.08)	92 (12)	LB (12%), MB (23%), OS (19%), OH (35%), ST (12%)
	**Injured (*n* = 5)**	29 (4)	2.05 (0.05)	105 (8)	LB (0%), MB (20%), OS (40%), OH (40%), ST (0%)
	**Complaints (*n* = 23)**	28 (5)	1.98 (0.08)	92 (12)	LB (13%), MB (22%), OS (17%), OH (35%), ST (13%)
	**No Injury (*n* = 21)**	27 (5)	1.97 (0.08)	89 (11)	LB (14%), MB (24%), OS (14%), OH (33%), ST (14%)
	**No Complain (*n* = 3)**	19, 22, 27	2.00, 2.04, 2.12	81, 95, 104	MB, OS, OH

*Abbreviations*: *LB* Libero, *MB* Middle blocker, *OS* Opposite spiker, *OH* Outside hitter, *ST* Setter

### Injuries

During the analysis period, 12 of the 41 athletes had a total of 28 injuries, with 13 injuries occurring in 2018 (in six athletes), eight injuries in 2019 (in seven athletes) and seven injuries in 2021 (in six athletes) (annual injury rate = 9/year). The chi-square test indicated no significant difference in the frequency of injuries per year (χ^2^ = 2.21, df = 2, *p* = 0.33). In 2018, a middle blocker sustained four injuries, another middle blocker had three injuries, and a setter and an outside hitter each sustained two injuries. In 2019, a middle blocker had two injuries, and in 2021, a middle blocker and an opposite spiker each sustained two injuries. Three injuries led to the athletes being removed from the team (two anterior cruciate ligament ruptures, and one wrist flexor tendon rupture).

The anatomical regions with the highest prevalence of injuries were the knee (195/1,000 athletes), followed by the ankle (122/1,000 athletes) (Table 2). Regarding the severity of injuries, 57% (*n* = 16) were classified as light, 25% (*n* = 7) as moderate, and 18% (*n* = 5) as severe. The average recovery time of the athletes was 10 days (1 to 58 days).

**Table 2:** Details of injuries presented by the athletes

Region *Type/Diagnosis*	Number of Injuries	Prevalence^a^ (Injuries/1,000 athletes)	Occurrence		Athlete Position
			**Match**	**Training**	
Knee	8	195 (88 to 349)	-	-	-
*Joint sprain/ligament tear*	3	73 (15 to 199)	1	2	- MB (1), OS (1), OH (1)
*Hoffitis*	2	49 (6 to 165)	1	1	- MB (1), OS (1)
*Baker’s cyst rupture*	1	24 (1 to 129)	0	1	- OH (2)
*Bursitis*	1	24 (1 to 129)	0	1	- OS (1)
*Tendinopathy*	1	24 (1 to 129)	0	1	- MB (1)
Ankle	5	122 (41 to 262)	-	-	-
*Joint sprain/ligament tear*	4	98 (27 to 231)	2	2	- MB (1), OH (1), ST (2)
*Bone contusion*	1	24 (1 to 129)	0	1	- OS (1)
Abdomen *– Muscle strain/rupture*	2	49 (6 to 165)	0	2	- MB (2)
Forearm *– Tendon rupture*	2	49 (6 to 165)	0	2	- OH (2)
Lower leg – *Muscle contusion*	2	49 (6 to 165)	1	1	- MB (2)
Lumbar-sacral spine	2	49 (6 to 165)	-	-	-
*Muscle contusion*	1	24 (1 to 129)	0	1	- OH (1)
*Unspecific*	1	24 (1 to 129)	0	1	- OH (1)
Thigh *– Muscle contusion*	2	49 (6 to 165)	1	1	- MB (2)
Elbow *– Tendinopathy*	1	24 (1 to 129)	0	1	- MB (1)
Face *– Bruise*	1	24 (1 to 129)	0	1	- ST (1)
Hip *– Muscle strain/rupture*	1	24 (1 to 129)	0	1	- MB (1)
Neck *– Muscle contusion*	1	24 (1 to 129)	1	0	- MB (1)
Shoulder *– Unspecific*	1	24 (1 to 129)	0	1	- MB (1)

^a^Prevalence and 95% confidence interval

*Abbreviations*: *LB* Libero, *MB* Middle blocker, *OS* Opposite spiker, *OH* Outside hitter, *ST* Setter

For the year 2018, an incidence of seven injuries per 1,000 h of competition, and two injuries per 1,000 h of training was obtained. Considering all analysed years, 21 injuries occurred during training Sect. (75%), and seven injuries occurred during matches (25%) (Table 3).

**Table 3:** Contingence table for injury frequency^a^ in each year, categorized by moment, age and position

	**2018**	**2019**	**2021**	**Total**
***by Moment***				
Match	4	3	0	7
Training	9	5	7	21
***by Age***				
≤ 23 years	1	2	0	3
> 23 years	12	6	7	25
***by Position***				
Libero	0	0	0	0
Middle blocker	7	5	2	14
Opposite spiker	0	1	3	4
Outside hitter	4	1	2	7
Setter	2	1	0	3

^a^Frequency in absolute values

Older athletes (> 23 years) presented a higher frequency of injuries compared to younger athletes (≤ 23 years) (Table 3). Among the athletes’ position, middle blocker had the highest injury frequency (14 injuries – 50%), followed by outside hitter (7 injuries – 25%), opposite spiker (4 injuries – 14%) and setter (3 injuries – 11%); no injuries were observed in the libero position (Tables 2 and 3).

### Complaints

During the analysis period, 38 of the 41 athletes reported a total of 402 complaints, with 140 complaints reported in 2018, 123 complaints in 2019 and 139 complaints in 2021. The chi-square test indicated no difference in the frequency of complaints per year (χ^2^ = 1.36, df = 2, *p* = 0.51). The annual complaint rate was 134 complaints per year, and normalised by athletes, the rate was three complaints per athlete per year. The complaints required 1,085 treatment sessions at a rate of 362 sessions per year.

**Fig. 1:**
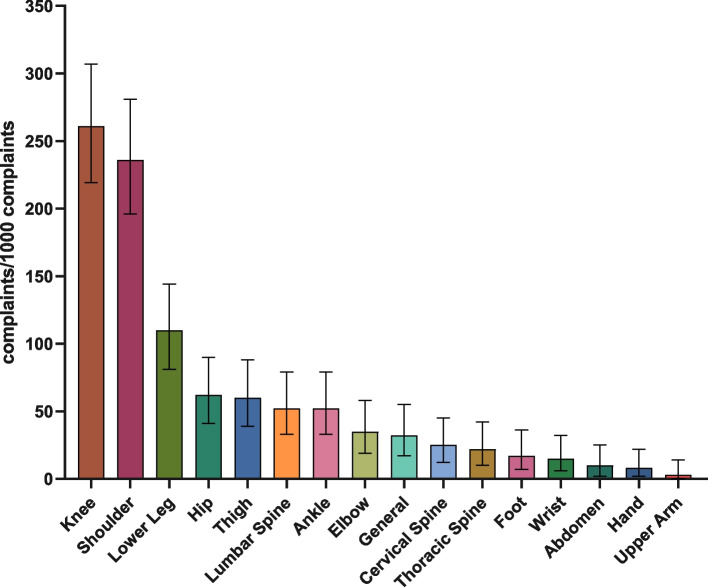
Prevalence of complaints according to anatomical regions (inner bars indicate 95% confidence interval)

**Fig. 2:**
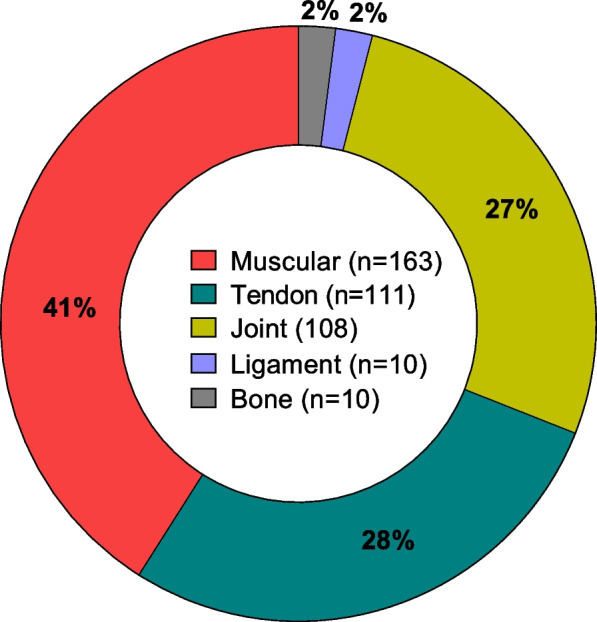
Characteristics of affected structures by complaints

Anatomical regions with the highest prevalence of complaints were the knees (261/1,000 complaints) and shoulders (236/1,000 complaints) (Fig. 1). Characteristics of complaints showed the highest prevalence for muscular complaints (41%), followed by tendinous (28%) and joint (27%) (Fig. 2).

**Fig. 3:**
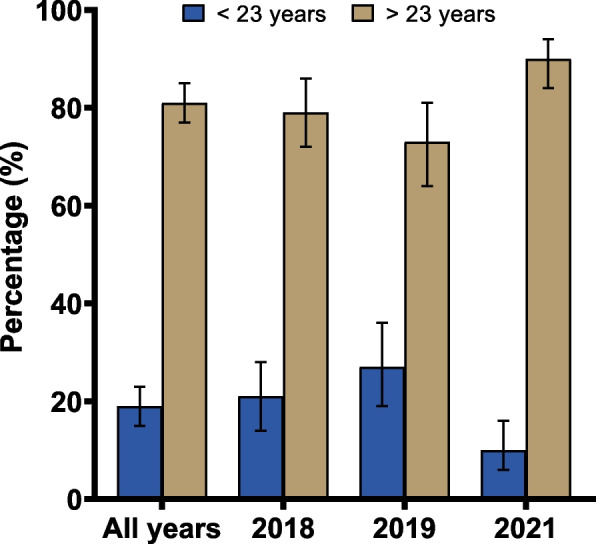
Frequency of complaints according to athletes’ age (inner bars indicate 95% confidence interval)

**Fig. 4:**
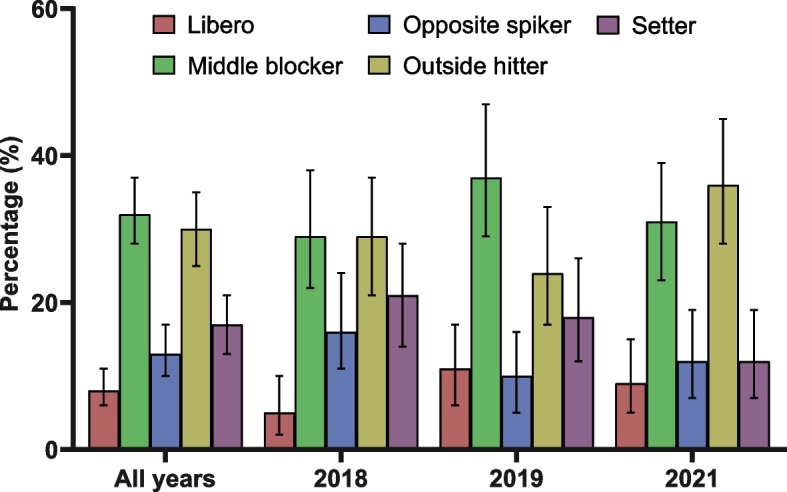
Frequency of complaints according to athletes’ position (inner bars indicate 95% confidence interval)

Older athletes (> 23 years) presented significant higher frequency of complaints compared to younger athletes (≤ 23 years) for all the analysed years (all years: χ^2^ = 155.47, df = 1, *p* < 0.01; 2018: χ^2^ = 48.03, df = 1, *p* < 0.01; 2019: χ^2^ = 26.42, df = 1, *p* < 0.01; 2021: χ^2^ = 88.64, df = 1, *p* < 0.01) (Fig. 3). The chi-square test indicated that there are significant differences in the frequency of complaints per athletes’ position (all years: χ^2^ = 91.18, df = 4, *p* < 0.01; 2018: χ^2^ = 27.86, df = 4, *p* < 0.01; 2019: χ^2^ = 32.00, df = 4, *p* < 0.01; 2021: χ^2^ = 43.43, df = 4, *p* < 0.01), being middle blocker and outside hitter the positions with the highest frequencies of complaint (Fig. 4). Considering all years, middle blocker had the highest frequency of complaints (130 complaints – 32%), followed by outside hitter (120 complaints – 30%), setter (68 complaints – 17%), opposite spiker (52 complaints – 13%) and libero (32 complaints – 8%) (Fig. 4).

## Discussion

The present study reported that injuries occurred more frequently during training sessions compared to matches. However, we observed a higher incidence of injuries during matches than during training sessions for the 2018 season. The regions most affected by injuries were the knee and ankle. We could consider the average recovery time short; the severity of injuries, most of which were light, likely influenced the short average recovery time. During the analysis period, almost all athletes presented complaints, which resulted in a high demand for the healthcare team, reflected in the high number of treatment sessions required to manage complaints. The knee and shoulder had the highest prevalence of complaints, with most complaints related to muscular regions. Athletes older than 23 years, middle blockers and outside hitters presented the highest frequencies of injuries and complaints.

We found that the vast majority of athletes reported complaints that did not keep them away from volleyball activities, but required attention and intervention by the healthcare team. The high frequency of complaints may indicate that the athletes were overloaded with activity. This is likely due to both the amount of training sessions and the accumulation of load from playing for domestic teams during regular season, without an adequate recovery interval. Moreover, considering that the complaints and discomforts are potential risk factors for injury occurrence [17–19], our results may indicate that most of the athletes were at risk of suffering an injury that would lead to time off from their activities. The agreement between regions with the highest prevalence of injuries and complaints suggests a likely relationship between them. For example, the knee was the region with the most complaints and injuries. These factors reinforce concerns related to prevention, particularly for specific subgroups that present a higher prevalence of injuries and complaints, such as athletes aged above 23 years and those playing as middle blocker or outside hitter [4, 5, 27]. While the Brazilian national team and other top teams incorporate prevention strategies into their physical training session, there remains a need for improvements in prevention programs. The literature is sparse in high-quality studies on effective preventive interventions for elite volleyball athletes [28, 29]. Despite the limited evidence, preventive interventions and preparation routines should continue as proactive alternatives for reducing complaints and injuries of athletes.

The knee was the body region most affected by injuries in the present study, accounting for 29% of the injuries, followed by the ankle, which accounted for 18% of the injuries. This pattern was not observed in previous studies, which found the ankle as the most affected body part in volleyball athletes, followed by the knee [5, 6]. Verhagen et al. [6] reported 41% of injuries in the ankle and 12% in the knee, and Bere et al. [5] reported 29% of injuries in the ankle and 15% in the knee. The difference between the present results and previous data is likely caused by the characteristics of the athletes. Verhagen et al. [6] included athletes from lower-level divisions who likely have a lower workload compared to elite athletes, and therefore, it is possible that the knee is less affected in these athletes. Bere et al. [5] reported data from world-class athletes, including junior and senior, male and female athletes. In this case, the difference is potentially explained by the characterization of an injury. In this study, an injury was defined as a musculoskeletal complaint that occurred during matches or training, regardless of whether this complaint caused absence from the sport activities or not [5]. Thus, we can infer that some injuries reported in the study by Bere et al. [5] may not have been actual injuries but rather discomforts that did not impact the ability of athletes to participate in sport activities. This reinforces the importance of using literature-based definitions to standardise the concept of injury, facilitating comparisons across different studies [22].

The results report that most of the complaints and injuries occurred in the lower limbs; this may be due to a high stress-load on the lower limbs during volleyball practice, resulting in a higher susceptibility of an overuse injury than other regions [30]. The data on the shoulder region showed an exception for the complaint and injury relationship observed in the present study. Although shoulder complaints were highly prevalent, only one injury occurred in this region. Perhaps, due to the essential role of the shoulder in the practice of volleyball, athletes are more alert to the slightest sign of discomfort and seek assistance to prevent an actual injury. Another potential explanation is related to the hypothesis that most shoulder pain is not disabling, as has been observed in other sports [31, 32]. Athletes with shoulder pain could adapt to the new condition and maintain their sports activities [33]. Future studies could address this issue by investigating the relationship between shoulder complaints and sports performance. A similar pattern may have occurred with the fingers of the hand. Previous studies reported that fingers are frequently affected by injuries in volleyball athletes [4, 5], which was not observed in the present study. The elite athletes did not sustain any finger injury during analysis period. However, we observed some complaints in this region. Therefore, as observed for the shoulder, we can suggest that pain or discomfort in the fingers is common among elite athletes and, in most cases, is not enough to cause them to be absent from their activities.

The incidence of injuries during training sessions observed in the present study is similar to previous data in volleyball, regardless if the athletes’ level [6, 11, 12]. Studies with athletes of the Norwegian Volleyball Federation [11], the second and third Dutch volleyball division [6] and the Greek Volleyball Federation [12] reported an incidence of approximately two injuries for every 1,000 h of training. For injuries during matches, we found a higher incidence in the present study compared to the previous studies [6, 11]. We verified the incidence of seven injuries for every 1,000 h of matches and previous studies indicated an incidence of approximately four injuries for every 1,000 h of matches [6, 11]. This difference may have multiple causes [3, 11, 13], but the specificity of elite athletes playing for national teams is likely the main factor for the higher incidence of injuries during matches. Participating on national teams is a life goal for most professional athletes; therefore, they may overcome physical limits during competitions, increasing the risk and occurrence of injuries.

The results on incidence and prevalence of injuries may be considered divergent. In the incidence analysis for 2018, the injuries occurred more frequently during matches than in training sessions. However, when considering the absolute number of injuries alone, we could affirm that athletes were injured more frequently during training sessions compared to matches. Since athletes spend a significantly greater amount of time in training compared to the duration of matches, the total number of training hours disperses the occurrence of injuries. Therefore, when normalising the data for the same amount of time, it becomes clear that athletes were injured more during matches than in training. Another important aspect to consider is the frequency of injuries per year. Although there is no difference in the proportion of injuries, we observed a higher frequency of injuries in 2018, which was nearly twice the frequency in 2021. In 2018, we observed that multiple athletes sustained more than one injury, with one athlete experiencing four injuries in that season. Thus, it is necessary to contextualize and interpret these results since, despite the higher frequency, the number of injured athletes remains similar across the three analysed years.

The most serious injury excluded the athlete for 58 days of practice, with an average of 10 days of absence. We can consider that 10 days is a short period off the activities and the characteristic of being an elite athlete likely contribute for a faster recovery. Even so, any absence directly affects a national team’s activities. This is especially important considering the limited period in which elite athletes are act for national teams, which reinforces the importance of the findings of the present study.

### Limitations

Although we followed up with our sample for three years, we were not able to report the injury incidence (injuries per played hours) during the entire Olympic cycle. Another potential limitation is related to the reporting of complaints in 2021 (the year of the Olympic Games). During this period, athletes may have concealed complaints due to the proximity of an important event, such as the 2021 Olympics. The COVID-19 pandemic also interfered with team activities, which may have given specificity to the analysed period. Furthermore, our results are only applicable to elite athletes, excluding amateur or professional athletes of a lower competition level. The high frequency of injuries and complaints in older athletes and those playing as middle blockers and outside hitters should be analysed with caution, considering that the majority of athletes playing for the Brazilian team are above 23 years old, and coaches usually select more athletes from these positions. Finally, in our study we did not quantify or qualify the intensity of complaints reported by athletes, which is a potential area for future investigations.

## Conclusion

Despite the low number of injuries, almost one-third of the athletes had injuries during the study period. In addition, almost all the athletes reported complaints during the follow-up period. Injuries and complaints were more prevalent in the knees. Therefore, preventive strategies should focus on the lower limbs, especially given the fact that it is a region commonly affected by overuse due to the fundamental demands of the sport. The epidemiology of complaints/injuries and the number of sessions to treat complaints are important predictive factors for managers to prepare themselves to assist these elite athletes in terms of staff and materials for this demand.

## Data Availability

The datasets used and analyzed during the current study available from the corresponding author on reasonable request.

## Abbreviations

FIVB: International volleyball federation
STROBE-SIIS: STROBE Sports Injury and Illness Surveillance
